# Clinical Features and Genetic Analysis of 20 Chinese Patients with X-Linked Hyper-IgM Syndrome

**DOI:** 10.1155/2014/683160

**Published:** 2014-08-20

**Authors:** Lin-Lin Wang, Wei Zhou, Wei Zhao, Zhi-Qing Tian, Wei-Fan Wang, Xiao-Fang Wang, Tong-Xin Chen

**Affiliations:** ^1^Department of Allergy and Immunology, Shanghai Children's Medical Center, Shanghai Jiao Tong University School of Medicine, Shanghai 200127, China; ^2^Department of Nephrology and Rheumatology, Shanghai Children's Medical Center, Shanghai Jiao Tong University School of Medicine, Shanghai 200127, China; ^3^Division of Allergy and Immunology, Department of Pediatrics, Virginia Commonwealth University, Richmond, VA 23298, USA; ^4^Division of Immunology, Institute of Pediatric Translational Medicine, Shanghai Jiao Tong University School of Medicine, 1678 Dongfang Road, Shanghai 200127, China

## Abstract

X-linked hyper-IgM syndrome (XHIGM) is one type of primary immunodeficiency diseases, resulting from defects in the CD40 ligand/CD40 signaling pathways. We retrospectively analyzed the clinical and molecular features of 20 Chinese patients diagnosed and followed up in hospitals affiliated to Shanghai Jiao Tong University School of Medicine from 1999 to 2013. The median onset age of these patients was 8.5 months (range: 20 days–21 months). Half of them had positive family histories, with a shorter diagnosis lag. The most common symptoms were recurrent sinopulmonary infections (18 patients, 90%), neutropenia (14 patients, 70%), oral ulcer (13 patients, 65%), and protracted diarrhea (13 patients, 65%). Six patients had BCGitis. Six patients received hematopoietic stem cell transplantations and four of them had immune reconstructions and clinical remissions. Eighteen unique mutations in *CD40L* gene were identified in these 20 patients from 19 unrelated families, with 12 novel mutations. We compared with reported mutation results and used bioinformatics software to predict the effects of mutations on the target protein. These mutations reflected the heterogeneity of *CD40L* gene and expanded our understanding of XHIGM.

## 1. Introduction

X-linked hyper-IgM syndrome (XHIGM; HIGM1; OMIM: 308230) is one type of primary immunodeficiency diseases (PIDs), resulting from defects in the CD40 ligand/CD40 signaling pathways leading to impairment of immunoglobulin isotype switching in B cells and characterized by recurrent infections in association with markedly decreased serum IgG, IgA, and IgE levels but normal or elevated serum IgM levels [1]. Patients with XHIGM usually develop symptoms by the first or second year of life. Over 50% of patients have chronic or intermittent neutropenia, often associated with oral ulcers. Opportunistic infections, especially* Cryptosporidium parvum *and* Pneumocystis jirovecii *infections, are common and prominent clinical feature of the patients. Besides the susceptibility to recurrent bacterial and opportunistic infections, these patients are prone to autoimmune manifestations, especially hematologic abnormalities, arthritis, and inflammatory bowel disease. Furthermore, an unusual susceptibility to liver, pancreas, or biliary tract tumours has been reported [2].

*CD40L *is the disease-causing gene of XHIGM [3–7], located in Xq26.3-Xq27.1 and containing five exons and four intervening introns. The corresponding translated protein CD154 (also called CD40L, gp39, TNFSF5, or TRAP) contains 261 amino acids and is mainly expressed on activated mature T cells. Its receptor, CD40, is expressed on a variety of cells, such as B cells, monocytes/macrophage, dendritic cells, endothelial cells, and epithelial cells [8, 9]. CD40-CD154 interactions provide a costimulatory signal for T cell activation and can lead to B cell proliferation and immunoglobulin class switching, affecting both cellular and humoral immunity [10, 11]. Therefore, XHIGM belongs to a combined immunodeficiency disease category.

XHIGM is the most common subtype of hyper-IgM syndromes (affecting about 70% of the patients) [12]. More subtypes of hyper-IgM syndromes have been gradually revealed. Diseases resulting from mutations in* AICDA *(ID: 57379),* CD40 *(ID: 958),* UNG *(ID: 7374)*, NEMO* (ID: 8517), or* NFKBIA *(ID: 4792) genes are all involved in the CD154-CD40- NF-*κ*B pathways and affected the class switch recombination (CSR), somatic hypermutation (SHM), or germinal center formation and form the subtypes of hyper-IgM syndromes. Mutations in the promoter of* CD40L* were also found [13]. The heterogeneity of hyper-IgM syndrome brings the challenge for diagnosis and accurate and reliable molecular testing methods are needed.

With diagnosed patients accumulated, people began to analyze the clinical features and mutation characteristics of these patients since 1992 [14]. A* CD40L* mutation database (http://structure.bmc.lu.se/idbase/CD40Lbase/index.php) has been founded and updated [15]. Retrospective research results of XHIGM patients in Europe [16], USA [17], Japan [18], and Latin America [19] were published. However, little information about Chinese XHIGM patients has been reported. In this study, we collected clinical data of Chinese XHIGM patients diagnosed and followed up in our center, reviewed their clinical and immune features, therapy, and response, and investigated the mutation characteristics, intending to increase our knowledge of this disease and finally improve the quality of life in these patients.

## 2. Methods 

### 2.1. Patients

Patients in present study were first diagnosed in hospitals affiliated to Shanghai Jiao Tong University School of Medicine or referred from other hospitals and followed up in Shanghai Children's Medical Center (also affiliated to Shanghai Jiao Tong University School of Medicine) during 1999–2013. A total of 28 candidate patients with HIGM phenotype were recruited. The inclusion criteria were low sera IgG and IgA (2 standard deviations below normal value for age), normal or elevated serum IgM, and compatible infectious events. Patients with secondary immunodeficiency conditions such as HIV or congenital rubella infections, immunosuppressive drug use, or neoplasms were excluded. Then CD154 expression on active T cells was detected by flow cytometry and sequences of* CD40L* gene were analyzed. A total of 20 patients with XHIGM were finally identified.

### 2.2. Data Collection

An informed consent was obtained from each patient's parent or guardian before enrollment in the study. Clinical data were collected from the patients' medical records, including initial clinical manifestation, onset age, diagnosis age, parental consanguinity, family history of immunodeficiency, recurrent infections, autoimmune diseases or malignancy diseases, vaccination and allergic history, complication, laboratory tests (including immunoglobulin level and lymphocyte subpopulations), and treatments. A total of 5 mL venous blood was collected from patients for pathogen screening and gene sequencing.

### 2.3. Molecular and Genetic Diagnosis

Detection of CD154 expression on activated CD4+ T cells was performed by flow cytometry (FACScan, Becton Dickinson, USA) using specific fluorescent-labeled monoclonal antibodies according to the method described previously [20].

Genomic DNA was isolated from heparinized peripheral blood by using the DP319 kit (Tiangen Biotech Co. LTD., China). The individual exons of* CD40L *gene, including exon-intron boundaries, were amplified by polymerase chain reaction (PCR) as previously described [21]. The system for PCR reaction contained 13.5 *μ*L ddH_2_O, 1 *μ*L primer for each direction, and 8.5 *μ*L 2 × Taq PCR MasterMix (containing 0.1 UTaq/*μ*L, 500 *μ*M dNTP, 20 mM Tris-HCl (pH 8.3), 100 mM KCl, and 3 mM MgCl2 (Tiangen Biotech Co. LTD., China), with total volume 25 *μ*L). Amplification conditions were 95°C for 10 minutes, then 40 cycles of 94°C for 30 seconds, 55°C for one minute, and 72°C for 30 seconds, followed by a final extension of 72°C for 2 minutes. The PCR products were electrophoresed in 1.5% agarose gel, purified, and sequenced. Mutations were investigated by alignment with standard sequence published in NCBI. We sequenced* CD40L* genes in 50 Chinese controls. Human SNP databases (dbSNP in NCBI and 1000 Genomes) were also searched to confirm the detected mutations.

### 2.4. Analysis Tools

Data were analyzed by SPSS statistical software (version 21.0, SPSS Inc., Chicago, IL). Median and range were used to present the characters of focused variable. Bioinformatics software PolyPhen-2 (http://genetics.bwh.harvard.edu/pph2/) and MutationTaster (http://www.mutationtaster.org/) were used to predict the effects of point mutations.

## 3. Results

### 3.1. Demographic Characteristics

From 1999 to 2013, 20 patients from 19 unrelated families (P2 and P3 are cousins) were diagnosed as XHIGM, considering the clinical manifestations, lab examination, family history, decreased CD154 expression, and the mutation of* CD40L* gene (Table 1). The median onset age of these patients is 8.5 months (range: 20 days–21 months). And the median of diagnosis lag is 50 months (range: 5 months–15 years). Half of the patients had positive family histories of previous sibling deaths at an early age and the diagnosis lag in them was much shorter than that in patients with negative family histories (31 ± 23 months* versus *85 ± 55 months, *P* = 0.015). Positive family history with early death in male members may help in diagnosis.

### 3.2. Clinical Manifestations

The most common symptoms of XHIGM patients were recurrent sinopulmonary infections (18 patients, 90%), neutropenia (14 patients, 70%), oral ulcer (13 patients, 65%), and protracted diarrhea (13 patients, 65%). Respiratory and gastrointestinal systems were more frequently affected. Skin (7 patients, 35%), bone and joint system (4 patients, 20%), central nervous system (3 patients, 15%), and urinary system (2 patients, 10%) were also affected (detailed in Table 1).

The median onset age of diarrhea was 12.0 months (range: 20 days to 20 months). Among them, two patients (P10, P11) were diagnosed with inflammatory bowel diseases (IBDs) under enteroscopes, and* Cryptosporidium* was isolated from the stool of P11. Fungus was isolated from the stool of P13. Pathogens in other patients were not identified.

Six patients had BCGitis (Table 2). The median onset time of adverse drug reactions was 2 months old (range: 50 days–7 months). Two of the patients received antituberculosis medicine treatments and recovered. The other four patients had severe local adverse events after BCG vaccinations, presenting ulceration >10 mm or suppurative lymphadenitis, and received surgeries to drain. Among them, P12 was reinfected by tubercle bacillus at 35 months old and recovered after antituberculosis drug therapy. Besides, another patient P14 suffered pulmonary tuberculosis at 8.5 years old, with restrictive pulmonary capacity and splenomegaly and lymphadenopathy. The patient received isoniazide and rifampicin treatment unregularly for nearly one year and developed tuberculous meningitis and died at 10 years old.

Some special manifestations deserved extra attention. P5 was infected by measles and developed subacute sclerosing panencephalitis (SSPE). P11 had* Cryptosporidium *infection, with protracted diarrhea and elevated liver enzyme. Moreover, this patient developed chronic kidney disease, presenting oliguria, edema, acidosis, increased creatinine concentration, and decreased glomerular filtration rate (GFR), lasting for over four months. Renal biopsy showed tubulointerstitial lesions and the patient received dialysis. P9 also developed chronic kidney disease, with oliguria, albuminuria, hematuria, increased serum creatinine and urea nitrogen concentration, and anemia for over three months. He also exhibited electrolyte disturbance (hypocalcemia and hyperphosphatemia) and convulsion. Ultrasound examination revealed diffuse kidney impairments.

### 3.3. Hematologic Findings

Anemia was found in seven patients (Table 3). Among them, P10 and P11 had IBDs and the chronic bloody purulent stools might partly cause the anemia. P9 and P11 had chronic kidney disease which may lead to renal anemia. P15 had pure red blood cell aplastic anemia demonstrated by bone marrow biopsy. Neutropenia was the most common hematologic complication, occurring in 70% of the patients (14 patients), 11 of which complicated with oral ulcer.

The median serum IgM concentration was 3.15 g/L (range: 0.74–11.7 g/L). Seventeen patients had elevated IgM levels and three patients had normal IgM levels. Serum IgG levels were uniformly decreased. IgA levels were reduced in the majority of patients.

Lymphocyte counts were generally normal. Three patients (P5, P9, and P11) exhibit significantly decreased CD4/CD8 ratio. They all had severe clinical symptoms and the decreased ratio reflected the impaired cellular immunity. P5 had measles infection, leading to SSPE, and died within one year. P11 had* Cryptosporidium *infection, with prolonged elevated liver enzyme and impaired renal functions. P9 also had severe manifestation, complicated with chronic renal failure.

### 3.4. Mutation Analysis

Eighteen unique mutations were identified in 20 patients from 19 unrelated families (P2 and P3 were cousins and they had the same mutation. Besides, P12 and P16 had the same mutation). Among the 19 families, we found 7 deletions, 6 missense mutations, 2 nonsense mutations, 2 splice site mutations, and 2 insertions (Table 4). The mutations were distributed throughout the entire gene, affecting all the domain of the protein, and exon 5 was the most frequently affected region (10/19, 52.6%). Detailed descriptions and analysis were in the Discussion section.

### 3.5. Therapy and Prognosis

Five patients had died (Table 1). P5 developed SSPE after measles virus infection at 15 years old and died within one year. P14 was infected by mycobacterium tuberculosis and died of tuberculous meningitis at 10 years old. P17 had severe diarrhea and pneumonia since 14 months and died at 2 years old. P3 and P13 received hematopoietic stem cell transplantations (HSCT) but suffered from severe infections and died at 22 months and 56 months, respectively. Three patients were lost in followups. The other twelve patients were alive till now, with the median age of 8 years old (range: 2–27 years old).

Fifteen patients (75%) received intravenous immunoglobulin (IVIG) treatment (Table 1). Eight patients could receive IVIG regularly (400 mg/kg/month) after diagnosis as HIGM. All of them were free of severe bacterial infections, although three of them still had recurrent oral ulcers. Seven patients could not receive full-dose IVIG regularly. Among them, one patient (P14) died of tuberculosis infection. Two patients still had recurrent bacterial infections (P2 had recurrent otitis media and perianal abscess. P12 had otitis media, pneumonia, and fever every half month). Three patients were lost in followup. The other five patients did not receive IVIG therapy, including the two patients who died from measles infection (P5) and severe diarrhea and pneumonia (P17).

Six patients received HSCTs and the median age at transplantation was 6 years old. Four patients received hematopoietic stem cells from HLA-identical unrelated donors and they had clinical remissions and were followed up from months to 6 years. The other two patients had HLA-half identical sibling donors and died from severe infections (Table 5).

## 4. Discussions

This study described the clinical features of Chinese XHIGM patients. Respiratory and gastrointestinal systems were more frequently affected. Skin, bone and joint system, central nervous system, and urinary system were also involved. In this study, two patients (P9 and P11) developed chronic kidney diseases. Cardiovascular disease, infections, and nonsteroidal anti-inflammatory drugs (NSAIDs) are accepted risk factors for causing chronic kidney disease [22]. P9 was born with congenital heart diseases (ventricular septal defect and patent ductus arteriosus). P11 had ulcerative colitis and received NSAIDs treatment. Both of the patients had severe recurrent infections because of immunodeficiency. These complications might partly contribute to the kidney involvement in these two patients. Few reports on XHIGM patients with chronic kidney diseases have been published till now. We should pay more attention to the urinary system function in further followups.

Opportunistic infections, especially* Cryptosporidium parvum *and* Pneumocystis jirovecii *infections, are common and prominent clinical feature of XHIGM patients [16, 17, 23]. Besides, infections caused by tubercle bacillus were detected [16, 17, 19]. A number of PIDs are reported to be susceptible to severe mycobacterial disease following vaccination with BCG, including severe combined immunodeficiency (SCID), chronic granulomatous disease (CGD), and Mendelian susceptibility to mycobacterial diseases (MSMD). Increased BCG infections are also reported in patients with hyperimmunoglobulin E syndrome (HIES) and XHIGM, with less prevalence and severity of BCG complications compared with the PIDs mentioned above [24]. In a European survey, only two of the 56 XHIGM patients present with tuberculosis [16]. However, in our study concerning 20 XHIGM patients, we found six patients had BCGitis during the first year of life. One of them was reinfected by tuberculosis at 35 months old. One more patient suffered pulmonary tuberculosis and tuberculous meningitis and died at 10 years old. In another cohort study focusing on Chinese patients, five (38%) of the 13 XHIGM patients had lymphoid tuberculosis after BCG vaccination [25], the relatively high incidence of which was very similar to ours. An Indian research group also reported that two patients with pulmonary tuberculosis and one patient with disseminated BCGiosis were found in the cohort study including seven XHIGM patients [26]. Global Tuberculosis Report 2013 by WHO pointed out that India and China had the largest number of tuberculosis cases, taking up to 26% and 12% of the global total, respectively [27]. In both China and India, BCG vaccine is given to every newborn after birth to prevent tuberculous meningitis and disseminated disease, while in Europe, where incidence and prevalence of tuberculosis are low, less than half of the European countries have nationally recommended BCG vaccination. The differences in tuberculosis prevalence and BCG coverage may partly contribute to the differences of mycobacteria complications in XHIGM patients.

Some recent discoveries may help to elucidate the susceptibility to mycobacterial infections in XHIGM patients. Hayashi's results indicated that CD40-CD154 signaling might be an important step in host immune response against* Mycobacterium avium *infection [28]. Klug-Micu et al. revealed that activation of monocytes via CD40L resulted in a Vitamin D-dependent antimicrobial activity against* Mycobacterium tuberculosis* [29]. IL12/23-IFN-*γ* axis is involved in defending against BCG infection [24, 30]. Jain et al. [31] demonstrated that activated T cells from XHIGM patients produced markedly reduced levels of IFN-*γ* and failed to induce antigen-presenting cells to synthesize IL-12. We should continue to observe the susceptibility to tuberculous infections in XHIGM patients and be concerned about the immunity affected by tuberculous infection. Furthermore, we found two patients with varicella-zoster virus infections, one patient with measles virus infections, one patient with fungus infections, and one patient with* Cryptosporidium *infection, which all indicated the defects in cellular immunity.

Mutations of* CD40L *geneare highly heterogeneous [18–20, 32]. CD40Lbase, which was last updated in 2011, contained 250 public entries. Besides, more mutations on* CD40L* gene were reported in other studies [19, 33]. Considering the published results, the common type of mutations in* CD40L* might be missense mutations, followed by deletion/insertion mutations, nonsense mutations, and splice site mutations (detailed in Table 6). In our results, missense mutations and deletion/insertion mutations were the main types, composing about 3/4 of the mutations.

Some possible mutation hotspots have been identified. Notarangelo et al. [15] pointed out that W140 represents a mutational hotspot. In Lee's study [32], four possible hotspots were identified that were Thr254, IVS1+1 g>t, IVS3+1 g>a/t, and IVS4+1 g>c. In the CD40Lbase, mutations were concentrated in some special positions; that is, mutations in C218, T254, and W140 have been identified in more than 10 patients, respectively, till now.

In this study, we identified two splice site mutations (P4: IVS2−1 g>a and P10: IVS4−2 a>g). Vargas-Hernández et al. [34] and García-Pérez et al. [35] separately reported a IVS3−1 g>a mutation, resulting in exon 4 skipping and a protein with 21-amino acid truncation. Weller et al. [36] reported an IVS2−1 g>t mutation, resulting in exon 3 skipping and forming a protein consisting of 107 amino acids. In our study, P4 mutated in 3′-acceptor splice sites, the same positions as the three cases, and it can be inferred that this mutation could also result in the skipping of exon 3. For the mutation in P10, it has been reported in 1994 [37]. Mutation in the acceptor site of intron 4 allowed utilization of a cryptic acceptor site and a few base pairs downstream in exon 5 and led to 8-nucleotides deletion in P10.

Six patients had missense mutations in this study. From data published in CD40Lbase (updated to 2011), the most frequent amino acid substitutions were Leu to Ser/Pro, Thr to Met, Gly to Arg, and Ala to Asp/Glu. Thusberg and Vihinen [38] reported that there were 10 invariant positions corresponding to the amino acids W140, L161, G167, Y169, Y172, L205, G226, G227, L231, and G257 in CD40L, according to sequence alignment and calculation. Disease causing mutations are typically located at conserved positions within a protein family, since these positions are usually essential for the structure and/or function of the protein [38]. In our study, the mutation in P6 (Gly167Arg) was previously reported in two Iranian patients [39]. Substitution from glycine to arginine introduced a larger side chain group and a positive charge, which would profoundly change structure and functions of the protein. The mutation in P20 (p.Trp140Arg) was reported as one of the “hotspots” and eleven patients in all had been reported to have this mutation until now. Trp140 is a large hydrophobic residue buried inside the protein while arginine had a large hydrophilic side chain with positive charge. The Trp140Arg substitution would obviously lead to destabilization of the structure.

Four missense mutations were novel. Mutation in P14 (p.Leu205Ser) changed the conserved hydrophobic residue into a hydrophilic residue. Proline was involved in the mutations in P11 (p.Pro10Ser) and P17 (p.Ala208Pro). Proline is the only amino acid forming a ring with the backbone and bends the main chain of the protein in a characteristic way. Proline is a known breaker of secondary structures. The substitution in the two patients would profoundly change structure and functions of the protein. A p.Ala208Asp mutation was identified in an XHIGM patient and reported in 2002 [40].

Effects of the four novel point mutations were analyzed by using bioinformatics software PolyPhen-2 [41] and MutationTaster [42]. PolyPhen-2 predicted probably damaging effects to the protein with a score over 0.99, respectively. MutationTaster predicts that mutations in P13, P14, and P17 were disease causing, with probability value over 0.98.

Two patients had nonsense mutations at codon 236 and codon 218, both of which were already reported [18, 36, 43]. Cys218 was involved in formation of a disulfide bridge and nonsense mutation (p.C218X) had been reported in at least twelve XHIGM patients and one carrier, usually associated with more severe clinical presentation.

Nine patients had insertion/deletion mutations, all changing the reading frames and leading to large changes of the protein structure. P12 and P16 were from unrelated families and they had the same mutation (g.2091_2094delTAGA, c.158_161delTAGA, p.Ile53fsX13). This mutation had been reported in at least another 6 unrelated patients and was the fifth common mutation position in the CD40Lbase. The 4 deleted nucleotides were very close to the acceptor splice site of intron 1 and were flanked by repeat sequences, suggesting that these mutations might be caused by slipped mispairing mechanisms. The frame shift deletion led to a premature stop codon within the extracellular unique domain. P2 and P3 were cousins and they had the same mutation (g.186delG). The deletion in exon 1 led to a premature stop codon in the transmembrane domain.

In conclusion, we retrospectively investigated the clinical, immunological, and molecular features of 20 Chinese XHIGM patients diagnosed and followed up in our center. We identified 18 unique mutations in* CD40L* in these patients, with 12 novel mutations. Various mutations reflected the heterogeneity of* CD40L* gene, and we should go further to investigate the relationship between genotype and phenotype.

## Figures and Tables

**Table 1:** Clinical features of patients with XHIGM phenotype.

Patients	Onset age (m)	Diagnosis age (m)	Diagnosis lag (m)	Status	Family history	Time for IVIG	HSCT	Oral ulcer	Otitis media	Recurrent sinopulmonary infections	Recurrent diarrhea	Neutropenia	Other significant events	Special pathogens
P1	18	84	66	L	(+)	7 y, IR		(+)∗		(+)				

P2	12	48	36	11 y	(+)	4 y, IR		(+)∗	(+)		(+)	(+)	Impetigo; perianal abscess	VZV

P3	6	12	6	D	(+)	1 y, R	(+)	(+)		(+)∗	(+)	(+)	Elevated liver enzyme; perianal abscess; eczema	

P4	15	20	5	L	(+)	19 m, IR			(+)	(+)	(+)		Encephalitis∗; elevated liver enzyme; sepsis; thrush; laryngitis	*Candida albicans*; *Pseudomonas aeruginosa *

P5	12	180	168	D		NA		(+)∗		(+)		(+)	SSPE; cerebral infarction; arthritis; eczema; urticaria	VZV; Measles; *Shigella *

P6	2	61	59	9 y	(+)	5 y, R	(+)	(+)		(+)	(+)	(+)	BCGitis∗ impetigo; sepsis; appendicitis; hepatitis;	HBV; *Pseudomonas aeruginosa *

P7	5	54	49	7 y		5 y, R			(+)	(+)		(+)	BCGitis∗	

P8	4	123	119	27 y		3 y, R	(+)	(+)		(+)	(+)∗	(+)	Arthritis	

P9	12	48	36	L	(+)	4 y, IR			(+)	(+)	(+)∗	(+)	Chronic kidney disease; arthritis;	

P10	9	60	51	9 y		2 y 5 m, R	(+)	(+)			(+)∗	(+)	Crohn's disease	

P11	12	192	180	21 y		NA			(+)	(+)	(+)∗		Ulcerative colitis; intestinal perforation; elevated liver enzyme; chronic kidney disease; arthritis	*Cryptosporidium *

P12	5	60	55	7 y	(+)	2 y 3 m, IR		(+)	(+)	(+)	(+)	(+)	BCGitis∗	*Mycobacterium tuberculosis *

P13	12	36	24	D		3 y, R	(+)	(+)		(+)∗	(+)	(+)	Hepatosplenomegaly	Fungus

P14	8	105	97	D		20 m, IR			(+)	(+)	(+)∗	(+)	Tuberculous meningitis	*Mycobacterium tuberculosis *

P15	6	77	71	11 y		7 y, IR		(+)	(+)	(+)∗			Perianal abscess; facial cellulitis; red blood cell aplastic anemia	

P16	0.67	60	59.33	6 y		3 y 4 m, R			(+)	(+)	(+)∗		BCGitis; asthma; urticaria	

P17	14	21	7	D	(+)	NA				(+)	(+)∗		BCGitis	

P18	21	49	28	5 y	(+)	NA		(+)	(+)	(+)∗		(+)		

P19	3	17	14	2 y	(+)	NA		(+)		(+)		(+)	BCGitis∗	

P20	6	41	35	4 y		3 y, R	(+)	(+)	(+)	(+)∗		(+)		

Total					**10**		**6**	**13**	**11**	**18**	**13**	**14**		

Note: (a) L: lost in followup; D: dead; R: regularly; IR: irregularly; NA: not available; y: year; m: month; IVIG: intravenous immunoglobulin; HSCT: hematopoietic stem cell transplantation; VZV: varicella zoster virus; HBV: hepatitis B virus. (b) ∗initial symptom.

**Table 2:** BCG vaccination-induced adverse drug reactions (ADR) and mycobacterium infections in XHIGM patients.

Patients	Ages	Performances	Treatments
P6	2 m	BCGitis (ipsilateral axillary lymphadenitis)	Surgery

P7	2 m	BCGitis (ipsilateral axillary lymphadenitis)	Surgery

P12	50 d	BCGitis (ipsilateral axillary lymphadenectasis)	Antituberculosis medicine (rifampicin, unregularly)
	35 m	Pulmonary tuberculosis	Antituberculosis medicine (isoniazide + rifampicin + VitB6), antibiotics

P16	7 m	BCGitis (ipsilateral axillary lymphadenitis)	Surgery

P17	2 m	BCGitis (ipsilateral axillary lymphadenitis)	Surgery

P19	3 m	BCGitis (ipsilateral cervical lymphadenectasis)	Antituberculosis medicine

P14	8.5 y	Pulmonary tuberculosis, restrictive pulmonary capacity, splenomegaly and lymphadenopathy	Antituberculosis medicine (isoniazide + rifampicin, unregularly), antibiotics, IVIG, thymosin
	10 y	Tuberculous meningitis	Antituberculosis medicine (isoniazide + rifampicin, unregularly)

Note: IVIG: intravenous immunoglobulin.

**Table 3:** Laboratorial features in XHIGM patients.

Patients (*n* = 20)	IgG (g/L)	IgM (g/L)	IgA (g/L)	Lowest neutropenia (/*μ*L)	Absolute lymphocyte (/*μ*L)	Hb (g/L)	PLT (∗1000/*μ*L)	CD3 (/*μ*L)	CD4 (/*μ*L)	CD8 (/*μ*L)	CD19 (/*μ*L)	CD4/CD8	CD16/56 (/*μ*L)
P1	<0.33	5.07	<0.07	3780	2450	115	320	NA	NA	NA	NA	NA	NA
P2	2.15	1.78	0.73	580	1498	90	479	1261	674	566	203	1.20	234
P3	0.39	0.92	0.23	1390	2110	111	444	1139	635	433	NA	1.32	NA
P4	0.08	9.48	<0.22	26000	3600	83	679	1116	572	486	756	1.18	1152
P5	<0.33	2.73	<0.07	900	3402	156	316	2007	700	1204	388	0.58	173
P6	<0.33	2	<0.07	139	1470	100	293	1021	786	182	392	4.32	42
P7	1.77	3.96	<0.24	360	5364	126	175	4076	2789	1180	912	2.36	375
P8	1.5	9.21	0.07	990	1285	100	184	1052	520	563	188	0.92	69
P9	<1.87	1.87	<0.03	306	4618	59	311	4248	1662	2424	222	0.68	111
P10	3.59	0.74	0.7	330	1350	81	243	999	729	189	346	3.86	28
P11	1.81	4.1	0.35	2214	1140	68	92	949	307	485	144	0.63	40
P12	0.32	3.65	<0.07	377	6200	120	673	4625	2263	1761	1389	1.29	174
P13	1.75	2.67	<0.24	190	5600	117	143	4178	2610	1349	1277	1.93	140
P14	2	3.56	0.52	200	1500	86	451	779	393	282	663	1.39	57
P15	1.21	11.7	0.24	1800	2778	19	325	2000	909	949	541	0.96	225
P16	0.45	2.24	0.55	2590	3360	117	335	2218	1277	605	706	2.14	370
P17	<0.33	1.98	<0.07	2000	6300	108	NA	2553	1323	1017	3024	1.24	504
P18	1.11	8.47	0.02	720	6140	112	476	4851	2579	1842	737	1.40	368
P19	0.199	0.77	<0.22	786	3144	96	633	2075	1100	1163	566	0.95	409
P20	0.27	3.8	0.42	440	3188	98	656	2295	1243	638	542	1.95	223

Note: NA: not available.

**Table 4:** Mutations in *CD40L* gene and CD40L expression in patients with XHIGM.

Patients (*n* = 20)	Genomic DNA mutation (NC_000023.10)	cDNA mutation (NM_000074.2)	Predicted effect on protein (NP_000065.1)	Affected domain	Exon/intron	CD40L expression	References
P1	g.175delC	c.175delC	p.Gln35ArgfsX36	TM	Exon 1	NA	
P2	g.186delG	c.186delG	p.Ser39GlnfsX48	TM	Exon 1	0.09%	
P3	g.186delG	c.186delG	p.Ser39GlnfsX48	TM	Exon 1	0.31%	
P4	g.6196g>a (IVS2-1g>a)	Skipping exon3	Skipping exon3		Intron 2	NA	
P5	g.8181_8182insGT	c.420_421insGT	p.Asp117ValfsX128	ECU	Exon 4	0.02%	
P6∗	g.10952G>C	c.499G>C	p.Gly167Arg	TNFH	Exon 5	0.27%	[39]
P7∗	g.11160C>A	c.707C>A	p.Ser236X	TNFH	Exon 5	0.75%	[36]
P8	g.11069_11072delCTCA	c.616_619delCTCA	p.Leu206GlufsX240	TNFH	Exon 5	0.80%	
P9∗	g.12090C>A	c.654C>A	p.C218X	TNFH	Exon 5	NA	[18, 19, 43]
P10∗	g.10861a>g (IVS4-2a>g)	c.482delTGTTACAG	Exon5 absent	TNFH	Intron 4	0.31%	[37]
P11	g.100C>T	c.100C>T	p.Pro10Ser	IC	Exon 1	4.55%	
P12∗	g.2091_2094delTAGA	c.158_161delTAGA	p.Ile53LysfsX65	ECU	Exon 2	0.01%	[18, 19, 44, 45]
P13	g.11220T>C	c.767T>C	p.Phe256Ser	TNFH	Exon 5	0.53%	
P14	g.11067T>C	c.686T>C	p.Leu205Ser	TNFH	Exon 5	1.78%	
P15	g.173_189delcccagatgattgggtca	c.173_189delcccagatgattgggtca	p.Thr34CysfsX42	TM	Exon 1	0.42%	
P16∗	g.2091_2094delTAGA	c.158_161delTAGA	p.Ile53fsLysX65	ECU	Exon 2	NA	[18, 19, 44, 45]
P17	g.11075G>C	c.694G>C	p.Ala208Pro	TNFH	Exon 5	NA	
P18	g.10986delG	c.605delG	p.Cys178PhefsX190	TNFH	Exon 5	NA	
P19	g.10882_10883insA	c.501_502insA	p.Gly144ArgfsX158	TNFH	Exon 5	NA	
P20∗	g.10871T>C	c.490T>C	p.Trp140Arg	TNFH	Exon 5	NA	[19, 38]

Note: ∗mutation that has been reported. IC: intracytoplasmic domain; TM: transmembrane region; ECU: extracellular unique domain; TNFH: tumor necrosis factor homology domain; NA: not available.

**Table 5:** Transplantation strategies for the six XHIGM patients.

Patients	Age at diagnosis (y)	Age at HSCT (y)	Pretransplantation status	Donors	Complications	Outcomes
P3	1	2	Oral ulcer, elevated liver enzyme, neutropenia	Haploidentical sibling	VOD, paralytic ileus, pulmonary infection, DIC	Died

P6	5	6.5	Gingivitis, neutropenia	HLA-identical unrelated donor	GVHD Grade I, ADH abnormal secretion.	9 y, clinical remission. Oral ulcer. Free of IVIG.

P8	10.25	21	Oral ulcer	HLA-identical unrelated donor	GVHD Grade III, sepsis (Brucella), CMV infection.	27 y, clinical remission. Pneumonia once a year within the first two years after HSCT. Free of IVIG.

P10	5	6	Oral and perianal ulcer, abdominal pain, diarrhea, fever, prolonged APTT, anemia	HLA-identical unrelated donor	GVHD Grade I, Hemorrhagic shock, VOD, CMV infection, diarrhea.	9 y, clinical remission. Free of IVIG.

P13	3	4.5	Oral ulcer, diarrhea, neutropenia	Haploidentical sibling	Sepsis	Died

P20	3.4	4.6	Oral ulcer, neutropenia	HLA-identical unrelated donor	GVHD Grade II, pulmonary infection.	4 y 10 m, clinical remission.

Note: HSCT: hematopoietic stem cell transplantation; VOD: hepatic venoocclusive disease; DIC: disseminated intravascular coagulation;

GVHD: graft-versus-host disease; ADH: antidiuretic hormone; IVIG: intravenous immunoglobulin.

**Table 6:** Mutation types in* CD40L* gene.

Reports	Year	Number of mutations	Missense (%)	Nonsense (%)	Deletion (%)	Insertion (%)	Splice site (%)
**This study**	**2013**	**19**	**31.6**	**10.5**	**36.8**	**10.5**	**10.5**
Nonoyama et al. [18]	1997	13	30.8	23.1	23.1	0	23.1
Seyama et al. [20]	1998	28	32.1	17.9	7.2	14.3	32.1
Lee et al. [32]	2005	61	18.0	24.6	21.3	11.5	24.6
Winkelstein et al. [17]	2003	54	22.2	18.5	24.1	11.1	24.1
**CD40Lbase [46]**	**2011**	**221**	**31.7**	**23.5**	**17.2**	**10.4**	**16.3**
Cabral-Marques et al. [19]	2014	26	34.6	26.9	11.5	7.7	11.5
